# Impact of Kidney Function on the Survival of Patients with Chagas Cardiomyopathy and Implantable Cardioverter Defibrillators

**DOI:** 10.3390/jcm14144862

**Published:** 2025-07-09

**Authors:** Fernanda Pinheiro Martin Tapioca, Luiz Carlos Santana Passos, Caio Cafezeiro, Willian Carvalho, Paulo Novis Rocha, Maria Gabriela Guimarães

**Affiliations:** 1 Nephrology Department, Ana Nery Hospital, Salvador 40301-155, Brazil; 2 Medical School, Bahiana School of Medicine, Salvador 40110-060, Brazil; 3 Post-Graduate Program in Medicine and Health, Federal University of Bahia, Salvador 40026-010, Brazil; 4 Cardiology Department, Ana Nery Hospital, Salvador 40301-155, Brazil; 5 Post-Graduate Program in Health Sciences, Federal University of Bahia, Salvador 40026-010, Brazil; 6 Department of Internal Medical and Diagnostic Support, Medical School of Bahia, Federal University of Bahia, Salvador 40026-010, Brazil

**Keywords:** chagas cardiomyopathy, heart failure, cardioverter defibrillators, kidney function

## Abstract

**Background/Objectives:** Impaired kidney function significantly increases mortality in recipients of implantable cardioverter defibrillators (ICDs). However, in the landmark studies evaluating ICDs and cardiac resynchronization therapy with a defibrillator (CRT-D) for the treatment of heart failure (HF) with a reduced ejection fraction (HFrEF), patients with Chagas cardiomyopathy (CC) have been underrepresented. This study aimed to determine whether kidney dysfunction has the same negative impacts on patients with ICDs or CRT-Ds and CC. **Methods**: We prospectively followed patients with CC and left ventricular ejection fractions (LVEFs) of ≤40% who underwent ICD or CRT-D implantation and had at least one prior creatinine measurement. The primary outcome was the survival rate during follow-up. Variables with a *p* of <0.10 from the univariate analysis were selected for inclusion in the Cox regression model. **Results**: A total of 343 patients were enrolled, with a median follow-up duration of 777 days. The mean age was 60.2 (±11.2) years. Fifty percent of patients were observed to have a New York Heart Association (NYHA) functional class of III, and the median left ventricular ejection fraction (LVEF) was 27% (22–32). Overall mortality events occurred in 113 (32.9%) participants during follow-up. Although the estimated glomerular filtration rate (eGFR) was significantly associated with survival in the univariate analysis [HR 0.98 (CI 95% 0.98–0.99), *p* = 0.007], it did not retain significance in the multivariate model [HR 0.99 (0.98–1.00), *p* = 0.138], which was adjusted for age, gender, atrial fibrillation (AF), body mass index (BMI), and the use of digoxin, furosemide, anticoagulants, and LVEF. **Conclusions**: Unlike other cardiomyopathies, impaired eGFR was not an independent predictor of mortality in this cohort of CC patients undergoing ICD or CRT-D implantation, possibly due to the distinctive pathophysiological mechanisms of the disease. These findings suggest that clinicians should not be discouraged from recommending CIEDs in patients with CC and moderately impaired kidney function, although further studies are warranted to assess outcomes in those with advanced CKD.

## 1. Introduction

Chagas cardiomyopathy (CC) is the second most common cause of heart failure (HF) in Latin America, with increasing prevalence in Europe and North America due to migration [1]. It is caused by chronic *Trypanosoma cruzi* infection, which promotes inflammation, cardiomyocyte death, and progressive fibrosis. These structural abnormalities, especially the formation of fibrotic reentry circuits, make CC a distinctly arrhythmogenic disease. Sustained ventricular tachycardia (VT) and subsequent ventricular fibrillation (VF) account for a majority of the sudden cardiac deaths in this population, even in the absence of severe systolic dysfunction [1,2]. Compared to other causes of dilated heart failure, CC is associated with a poorer prognosis, primarily due to the elevated risk of malignant arrhythmias [3,4].

In this context, cardiac implantable electronic devices (CIEDs)—particularly implantable cardioverter defibrillators (ICDs) and cardiac resynchronization therapy with defibrillators (CRT-Ds)—are essential tools for managing CC patients at high arrhythmic risk [4,5].

ICDs and CRT-Ds have consistently demonstrated survival benefits in patients with heart failure with reduced ejection fractions (HFrEF) in landmark clinical trials and are considered core components of guideline-directed medical therapy (GDMT) [6]. GDMT includes beta-blockers; renin-angiotensin-aldosterone system inhibitors (RAASi) such as angiotensin-converting enzyme inhibitors (ACEi), angiotensin receptor blockers (ARB), or angiotensin receptor-neprilysin inhibitors (ARNi); mineralocorticoid receptor antagonists (MRAs); and sodium-glucose cotransporter 2 inhibitors (SGLT2i). However, chronic kidney disease (CKD) remains a barrier to optimal HF management due its potential to worsen CKD and the increased risk of hyperkalemia associated with RAASi use [7]. Additionally, in patients with ICD, it has been demonstrated that even a 10 mL/min/1.73 m^2^ reduction in estimated glomerular filtration rate (eGFR) is associated with a substantial increase in mortality [8].

The cardiorenal interaction in HFrEF is complex and mutually reinforcing, shaped by overlapping neurohormonal, hemodynamic, and inflammatory pathways [9]. In ischemic cardiomyopathy (ICM), CKD exacerbates mortality through shared metabolic and inflammatory mechanisms, including endothelial dysfunction, oxidative stress, and accelerated atherosclerosis progression [10]. Conversely, CC’s pathophysiology is predominantly characterized by malignant arrhythmias rather than ischemic or systemic inflammatory processes. This distinction raises the possibility that renal dysfunction may not carry the same adverse prognostic impact in CC patients as it does in patients with other cardiomyopathies.

We hypothesized that impaired kidney function is not independently associated with mortality in CC patients receiving ICD or CRT-D implantation due to the disease’s uniquely arrhythmogenic substrate and overall poor prognosis. This study aimed to evaluate the prognostic impact of renal function on survival in real-world CC patients undergoing high-value CIED implantation in a middle-income, endemic setting.

## 2. Materials and Methods

### 2.1. Study Design and Population

We conducted a prospective observational study that included adult patients (≥18 years) receiving care at the HF Clinic of a tertiary hospital in Salvador, Bahia, Brazil, between June 2017 and December 2023. The study cohort comprised individuals with a prior diagnosis of Chagas disease (CD) and HFrEF, defined as a left ventricular ejection fraction (LVEF) of ≤40% on transthoracic echocardiography, who were consecutively referred for ICD or CRT-D implantation. The diagnosis of CD was confirmed through two independent serological tests—an enzyme-linked immunosorbent assay (ELISA) or indirect immunofluorescence (IFI). Other echocardiographic findings (beyond reduced ejection fraction) or electrocardiographic abnormalities, such as right bundle-branch block, were not required for inclusion to ensure a broad CC cohort reflective of real-world clinical practice, but they were recorded when present. The estimated glomerular filtration rate (eGFR) was calculated using the 2021 CKD-EPI equation, which incorporates age, sex, and serum creatinine levels [11].

The exclusion criteria included the absence of an eGFR measurement prior to device implantation, a prior history of any CIED, or patient refusal to undergo the procedure or sign the informed consent form.

This study was approved by the Institutional Review Board of Hospital Ana Nery, Brazil (protocol number 1.421.936) and was conducted according to the ethical principles of the Declaration of Helsinki. Written informed consent was obtained from all participants before enrollment.

### 2.2. Procedures

All study participants underwent CIED implantation at our center. Before the procedure, each patient had a clinical evaluation at the HF Clinic and Electrophysiology Center for pre-procedural planning. Laboratory data relevant to this study were collected and recorded during this visit. All patients received either an ICD or a CRT-D, both manufactured by Medtronic (Minneapolis, MN, USA), with implantation performed by the interventional electrophysiology team at Ana Nery Hospital. The procedure was conducted under local anesthesia following standard implantation protocols.

### 2.3. Follow-Up and Endpoints

The primary outcome of this study was the survival rate during the follow-up period. After hospital discharge, patients were monitored by a multidisciplinary team at the HF clinic. The frequency of follow-up visits was determined at the attending physician’s discretion based on clinical judgment. Mortality events that occurred during subsequent hospitalizations were systematically recorded. For patients who did not return for their scheduled follow-up, telephone contact was initiated to assess the clinical status and identify any adverse outcomes. In cases where death occurred at an external facility, family members reached the HF team and, whenever possible, official death certificates were requested to verify the circumstances of the event.

### 2.4. Statistical Analysis

Continuous variables are presented as means ± standard deviations (SDs) for normally distributed data or as medians and interquartile ranges (IQRs) for non-normally distributed data. Normality was assessed using the Shapiro–Wilk test. Categorical variables are reported as absolute frequencies and their corresponding percentages.

To evaluate factors associated with survival outcomes, we first performed univariate analyses using a Cox proportional hazards regression for each independent variable. Variables with a *p*-value of < 0.10 in the univariate analysis were subsequently included in a multivariate Cox regression model to identify independent predictors of mortality. Age and sex were preselected as covariates for inclusion in the final model based on their known prognostic relevance. The hazard ratio (HR), reported with a 95% confidence interval (CI), was used for the survival analyses. Statistical significance was set at a two-tailed *p*-value of <0.05.

All statistical analyses were performed using IBM SPSS Statistics (version 25.0, IBM Corp., Armonk, NY, USA) and RStudio (version 2024.04.1+748, R Foundation for Statistical Computing, Vienna, Austria).

## 3. Results

**Figure 1:**
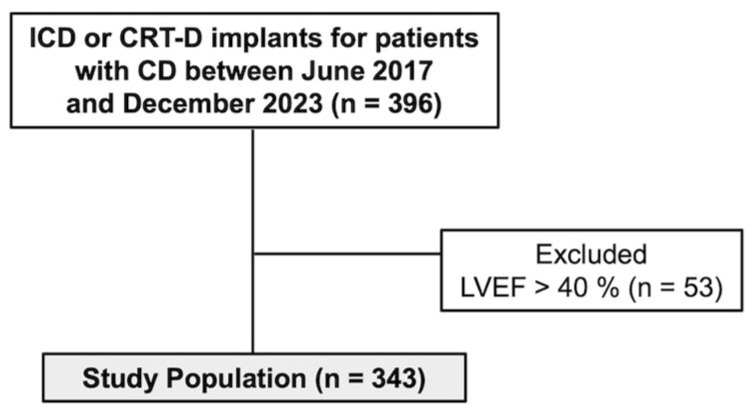
Flowchart of Patient Selection. Abbreviations: CD, Chagas Disease; ICD, implantable cardioverter defibrillator; CRT-D, cardiac resynchronization therapy with defibrillator; LVEF, left ventricular ejection fraction.

Between June 2017 and December 2023, 396 patients with CC were evaluated for ICD or CRT-D implantation at our center. Of these, 53 were excluded from the study due to having an LVEF of >40%. Ultimately, 343 patients were included (Figure 1). None of the patients were lost to follow-up during the study period.

The baseline clinical characteristics are presented in Table 1. Among the 343 patients, 225 (65.6%) were male, with a mean age of 60.2 (±11.2) years. Of these, 158 (46.1%) underwent CRT-D implantation, and 185 (53.9%) received ICDs, with no significant baseline differences in LVEF [median 26% (IQR 21–32) vs. 28% (IQR 23–32), respectively] or eGFR values between the groups [median 64.5 mL/min/1.73 m^2^ (IQR 47–81) vs. 67 mL/min/1.73 m^2^ (IQR 55–81 mL/min/1.73 m^2^), respectively]. The overall median eGFR was 66 mL/min/1.73 m^2^ (IQR 51–8166 mL/min/1.73 m^2^), with 30.0% of patients having CKD Stage 3 or worse (eGFR < 60 mL/min/1.73 m^2^). The overall median LVEF was 27% (IQR 22–32), and 174 (50.7%) patients presented with a New York Heart Association (NYHA) functional class of III, while 38 (11.1%) had a NYHA class of IV. The median follow-up period was 777 days.

High adherence to GDMT for HFrEF was observed in the study cohort. RAASis (including ACEi/ARB or ARNi) were prescribed to 84.3% of the patients. Beta-blockers were administered to 83.7% of the cohort, while spironolactone was given to 77.3%. Furosemide was prescribed to 72.0% of the patients. Amiodarone was used in 36.4% of the population, and 14.0% received digoxin. Anticoagulation therapy, which included vitamin K antagonists or direct oral anticoagulants, was prescribed to 34.1% of the patients (Table 2).

**Figure 2:**
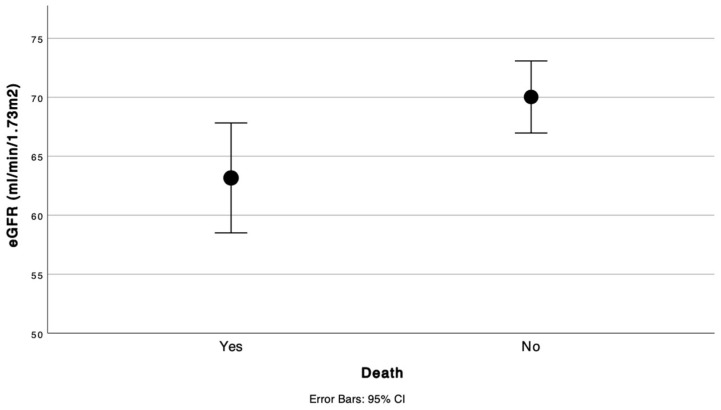
Estimated glomerular filtration rate according to all-cause mortality. eGFR, estimated glomerular filtration rate; CI, confidence interval.

During the follow-up period, 113 (32.9%) patients died. In the univariate Cox regression analysis, markedly traditional variables were not significantly associated with mortality, such as diabetes, previous infarction, or NYHA classification. Otherwise, several variables were significantly associated with overall survival (Table 3). AF doubled the mortality risk (HR 2.13, 95% CI 1.47–3.09, *p* < 0.001). Furosemide use (HR 1.96, 95% CI 1.21–3.18, *p* = 0.006) and anticoagulation therapy (HR 1.76, 95% CI 1.21–2.56, *p* = 0.003) were also significantly associated with increased mortality. BMI was inversely correlated with mortality, suggesting a protective effect of a higher BMI (HR 0.92, 95% CI 0.88–0.97, *p* = 0.001). LVEF was correlated inversely with mortality (HR 0.94, 95% CI 0.92–0.97, *p* < 0.001), indicating that lower LVEF values predicted poorer outcomes. Additionally, eGFR was linked to mortality (HR 0.98, 95% CI 0.98–0.99, *p* = 0.007), suggesting that declining renal function increased mortality risk (Figure 2).

**Figure 3:**
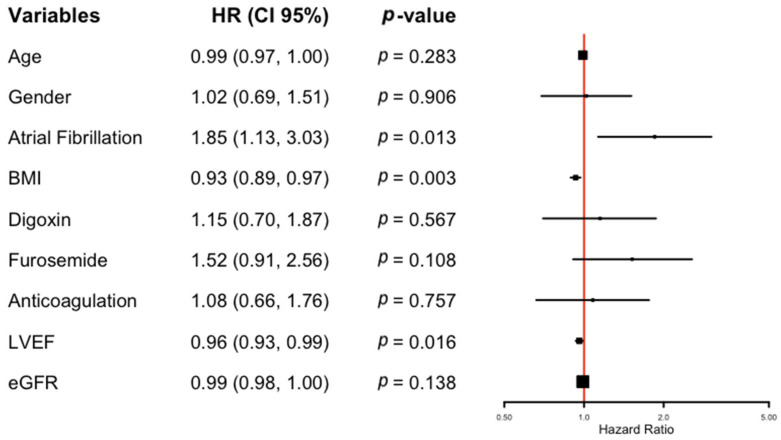
Multivariate Cox regression analysis for survival. HR, hazard ratio; CI, confidence interval; BMI, body mass index; LVEF, left ventricular ejection fraction; eGFR, estimated glomerular filtration rate.

In a multivariate Cox regression model, AF (HR 1.85, 95% CI 1.13–3.03, *p* = 0.013), BMI (HR 0.93, 95% CI 0.89–0.97, *p* = 0.003), and LVEF (HR 0.96, 95% CI 0.93–0.99, *p* = 0.016) remained independent predictors of mortality (Figure 3). However, other variables that were significant in the univariate analysis, such as furosemide use, anticoagulation, and digoxin, did not retain statistical significance in the multivariable model. Importantly, eGFR was not an independent predictor of mortality after adjusting for confounding factors (HR 0.99, 95% CI 0.98–1.00, *p* = 0.138), suggesting that kidney function alone did not significantly impact survival in this population of patients with CC undergoing CIED implantation.

**Figure 4:**
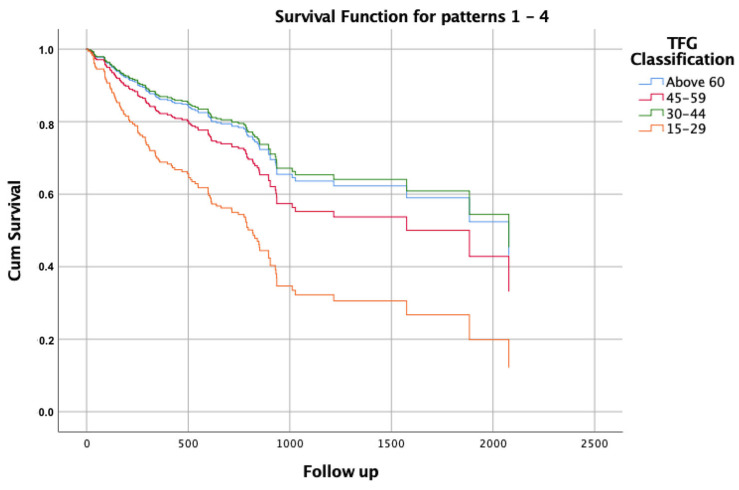
Kaplan–Meier survival estimates for cumulative survival according to the following four eGFR categories: >60 mL/min/1.73 m^2^ (blue), 45–59 (red), 30–44 (green), and 15–29 (orange). The patients with severely reduced eGFR (15–29) had higher mortality rates over time (log-rank test—*p* = 0.022). In a multivariate model adjusted for age, gender, BMI, LVEF, and use of digoxin, furosemide, atrial fibrillation, and anticoagulation therapy, the results showed an HR 2.5 (95% CI 1.16–5.37; *p* = 0.019).

To further investigate, we conducted an additional multivariate Cox regression model, using the same variables in the prior model (age, gender, BMI, LVEF, digoxin, furosemide, anticoagulation, and atrial fibrillation), but we stratified the stages of CKD (Figure 4) [12]. In this analysis, there were 8 deaths out of the 12 patients with eGFR measurements of 29−15 mL/min/1.73/m^2^**,** which demonstrated the significantly increased mortality rate in this stratum (HR 2,5 95% CI 1.16–5.37, *p* = 0.019).

## 4. Discussion

The present study is the largest cohort of CC patients with ICDs (with or without CRT-D) that has attempted to investigate the relationship between expensive CIEDs and kidney function in CC patients.

We evaluated the impact of impaired kidney function on survival in 343 patients with severe HFrEF secondary to CC who underwent ICD or CRT-D implantation. In our population, moderately decreased eGFR did not significantly affect survival rate, although LVEF, BMI, and AF were identified as significant risk factors in the multivariate analysis. This finding contrasts with other cardiomyopathies, where even a mild reduction in eGFR is associated with increased mortality. For instance, a study by Kato et al. demonstrated that a one-year decline of 10 mL/min/1.73 m^2^ in eGFR resulted in a nearly two-fold increase in mortality risk among heart failure patients (hazard ratio: 1.97; 95% CI: 1.45–2.69; *p* < 0.001) [13].

The overall mortality rate in our cohort was 32%, which was higher than those reported in previous studies [3,4,14,15]. Our population was at a higher risk of death based on clinically relevant parameters, such as a high NYHA score and a mean LVEF of 27% [16].

Although kidney disease plays a well-established role in the survival of HFrEF patients, especially in non-Chagas recipients of CIEDs, we did not see the same pattern in our population [17]. Our findings align with those of Nakazone et al., who assessed CKD and anemia as risk factors for mortality in a CC population [18]. In this previous study, the patients with impaired renal function were older, had larger right ventricular diameters, and required pacemakers more often, indicating more advanced cardiomyopathy. However, they did not find an independent association between either CKD or anemia and poorer survival. Likewise, Ferreira et al. studied 296 HF patients, mostly with CC, to evaluate the effects of kidney failure and anemia [19]. Despite a relevant proportion of participants having an eGFR of below 60 mL/min/1.73 m^2^, kidney impairment was not linked to worse outcomes.

Notably, the median eGFR in our cohort was 66 mL/min/1.73 m^2^, indicating only mild kidney dysfunction. This could be attributed to the clinical tendency to withhold CIED implantation in patients with more advanced CKD. In the small sample of 12 patients with eGFR values of 15–29 mL/min/1.73 m^2^, the high event rate (66%) produced an adjusted HR of 2.50, with a nominally significant *p*-value (0.019). This may be explained by the decreased responsiveness to ICD and CRT in advanced CKD due to higher defibrillation threshold/non-capture and increased myocardial fibrosis [17]. However, we recognize that the confidence interval is wide and the result is statistically fragile (one additional survivor would render it non-significant).

Additionally, we observed an unexpectedly high 80% adherence to GDMT, which may have provided a protective effect not observed in past studies with more severe CKD [20]. The growing use of GDMT, which has been uncommon in other studies of real-world patients, may have mitigated some adverse effects of mild CKD, potentially explaining the lack of eGFR significance.

The existing literature on kidney diseases and HFrEF predominantly includes patients with ICM, neglecting CC as a significant HF etiology [1,21,22]. ICM and CKD share common comorbidities such as diabetes, hypertension, and dyslipidemia, contributing to a vicious cycle where CKD worsens HF prognosis, and cardiovascular events are more severe in patients with kidney impairment [10,20]. Patients with CC do not usually present with the classical context of the cardiovascular-kidney-metabolic syn-drome and its pathophysiology, which might reduce the clinical impact of a lower eGFR on its outcomes [23]. Addressing this matter in this specific population of patients may facilitate clinical decision-making and encourage further studies.

In our cohort, we found the so-called “obesity paradox”, where mild to moderate obesity is associated with a lower mortality amongst patients with HF [24]. There are many explanations for this phenomenon, such as an earlier diagnosis of HF in obesity patients, and a high BMI may indicate less-advanced disease. This observation warrants further investigation in CC cohorts.

Future research should enhance risk stratification models for CC patients with CIEDs by incorporating novel biomarkers and cardiovascular magnetic resonance to predict outcomes beyond eGFR more effectively [25,26]. Multi-center studies are essential for assessing whether tailored approaches, including expanded indications for ICD implantation, could benefit patients with CC and impaired renal function. Moreover, the effects of newer HFrEF therapies, such as SGLT2i and ARNI, on this population remain largely unexplored and require further investigation [8,27].

This study had some limitations. It was an observational, prospective, single-center study, and no formal statistical power calculation was performed. Moreover, although the patients were actively encouraged to attend post-ICD evaluations, the variable follow-up intervals—determined at the physician’s discretion—may have introduced surveillance bias. Notably, there were virtually no missing data or losses to follow up.

Given the limited representation of patients with markedly reduced eGFR, the study may have been underpowered for detecting incremental hazards where renal function was modelled as a continuous covariate in the survival analysis. An association with excess mortality became evident only after stratifying eGFR by Kidney Disease: Improving Global Outcomes (KDIGO) stages, yet the broad confidence intervals around this categorical effect necessitated cautious interpretation. Notwithstanding these constraints, the present investigation constitutes the most comprehensive longitudinal dataset on Chagas cardiomyopathy recipients of CIEDs to date, thereby filling a critical evidentiary gap for this under-studied cohort

Furthermore, serum creatinine levels were assessed only at the time of device implantation, which may not have adequately captured long-term renal function trajectories [8,27]. Finally, the study was initiated in 2017, prior to the widespread implementation of SGLT2 inhibitors and ARNI as standard therapies for HFrEF [28,29].

Further multicenter prospective studies are needed to investigate cardiorenal interaction in CC patients, since even with a large population, our study may not have the statistical power to detect a significant difference between eGFR levels and hard outcomes.

## 5. Conclusions

In conclusion, in a population of patients with severe CC undergoing ICD or CRT-D implantation, moderate eGFR did not significantly impact overall survival, unlike the known pattern of other cardiomyopathies. Therefore, the bedside decision to implant these CIEDs in CC patients with HFrEF should not be withheld on the basis of moderate kidney dysfunction. However, further prospective studies are warranted to assess outcomes in patients with advanced chronic kidney disease.

## Figures and Tables

**Table 1:** Baseline characteristics of the patients (*n* = 343).

Variable	
Male (%)	225 (65.6)
Age, years (± SD)	60.2 (11.2)
CRT-D (%)	158 (46.1)
ICD (%)	185 (53.9)
Hypertension (%)	226 (65.9)
Diabetes (%)	85 (24.8)
Atrial fibrillation (%)	106 (30.9)
Prior stroke (%)	69 (20.1)
Prior myocardial infarction (%)	49 (14.3)
BMI kg/m^2^ (IQR)	23 (20–27)
Maggic SCORE (IQR)	13.4 (10.2–19.1)
Ejection fraction,% (IQR)	27 (22–32)
NYHA III (%)	174 (50.7)
NYHA IV (%)	38 (11.1)
eGFR, mL/min/1.73/m^2^ (IQR)	66 (51–81)
Median follow-up, days (IQR)	777 (488–904)

Abbreviations—SD, standard deviation; CRT-D, cardiac resynchronization therapy with a defibrillator; ICD, implantable cardioverter defibrillator; BMI, body mass index; NYHA, New York Heart Association; eGFR, estimated glomerular filtration rate; IQR, interquartile range.

**Table 2:** Profile of the prescribed drugs.

Drug Class	*n*, (%)
RAASi ^+^, *n*, (%)	289 (84.3)
Beta blockers, *n*, (%)	287 (83.7)
Spironolactone, *n*, (%)	265 (77.3)
Hydralazine + nitrate, *n*, (%)	60 (17.5)
Furosemide, *n*, (%)	247 (72.0)
Amiodarone, *n*, (%)	125 (36.4)
Digoxin, *n*, (%)	48 (14.0)
Anticoagulation ^$^, *n*, (%)	117 (34.1)

Abbreviations: RAASi ^+^, renin-angiotensin-aldosterone system inhibitors, such as angiotensin II receptor blockers, angiotensin-converting enzyme inhibitors, or sacubitril-valsartan. ^$^, either vitamin K antagonists or direct oral anticoagulants.

**Table 3:** Univariate and multivariate Cox regression analysis of the variables associated with overall survival.

	Univariate Analysis		Multivariate Analysis	
	HR (CI 95%)	*p*-Value	HR (CI 95%)	*p*-Value
Age	1.00 (0.98–1.02)	0.657	0.99 (0.97–1.00)	0.283
Gender	0.93 (0.63–1.37)	0.730	1.02 (0.69–1.51)	0.906
Atrial fibrillation	2.13 (1.47–3.09)	<0.001	1.85 (1.13–3.03)	0.013
BMI	0.92 (0.88–0.97)	0.001	0.93 (0.89–0.97)	0.003
Digoxin	1.59 (1.00–2.54)	0.049	1.15 (0.70–1.87)	0.567
Furosemide	1.96 (1.21–3.18)	0.006	1.52 (0.91–2.56)	0.108
Anticoagulation	1.76 (1.21–2.56)	0.003	1.08 (0.66–1.76)	0.757
LVEF	0.94 (0.92–0.97)	<0.001	0.96 (0.93–0.99)	0.016
eGFR	0.98 (0.98–0.99)	0.007	0.99 (0.98–1.00)	0.138
Prior MI	0.93 (0.55–1.59)	0.809	-	-
Diabetes	0.94 (0.61–1.46)	0.801	-	-
NYHA			-	-
-III	1.00 (0.68–1.47)	0.983	-	-
-IV	1.50 (0.89–2.54)	0.126	-	-

Abbreviations: HR, hazard ratio; CI, confidence interval; BMI, body mass index; LVEF, left ventricular ejection fraction; eGFR, estimated glomerular filtration rate; MI, myocardial infarction; NYHA, New York Heart Association.

## Data Availability

The data supporting this study’s findings are not publicly available due to ethical and legal restrictions as they contain information that could compromise patient confidentiality. Access to the dataset may be granted upon reasonable request to the corresponding author.

## Abbreviations

ACEi	Angiotensin-converting enzyme inhibitors
AF	Atrial fibrillation
ARB	Angiotensin II receptor blockers
ARNi	Angiotensin receptor-neprilysin inhibitors
BMI	Body mass index
CC	Chagas cardiomyopathy
CD	Chagas disease
CIED	Cardiac implantable electronic devices
CI	Confidence interval
CKD	Chronic kidney disease
CRT	Cardiac resynchronization therapy
CRT-D	Cardiac resynchronization therapy defibrillator
eGFR	Estimated glomerular filtration rate
HR	Hazard ratio
ICD	Implantable cardioverter defibrillator
ICM	Ischemic cardiomyopathy
IQR	Interquartile range
GDMT	Guideline-directed medical therapy
HF	Heart failure
HFrEF	Heart failure with reduced ejection fraction
LVEF	Left ventricular ejection fraction
NYHA	New York Heart Association
RAASi	Renin-angiotensin-aldosterone system inhibitors
SCD	Sudden cardiac death
SD	Standard deviation
SGLT2i	Sodium-glucose cotransporter 2 inhibitors
